# Between Many Rocks and Hard Places

**DOI:** 10.5005/jp-journals-10071-23228

**Published:** 2019-08

**Authors:** Manu Varma MK, Bhuvana Krishna, Sriram Sampath

**Affiliations:** 1-3 Department of Critical Care Medicine, St. John's Medical College and Hospital, Bengaluru, Karnataka, India

## Abstract

**How to cite this article:** Varma MMK, Krishna B, Sampath S. Between Many Rocks and Hard Places. Indian J Crit Care Med 2019;23(8):388.

Dear Sir,

The points raised in the letter of Bharat Kumar and colleagues are relevant and capture the difficulties of research in low and middle income countries (LMIC). The development and deployment of a regional registry is a progressive step and such steps should be encouraged and supported. Intensivists in India are attempting to move away from traditional practices, succinctly described by Berger as a “Cottage industry based on anecdotal experience and characterized by enormous practice variation”.^1^ The goal of creating a scientific ecosystem and the path to reliability and quality are further frustrated by the difficulties of rigorous randomized controlled trials or the known biases of observational research.

Bharat Kumar and colleagues have pointed out that their regional registry: Indian Registry of IntenSive care (IRIS) reduces the data collection burden by reducing the number of mandatory details which have to be filled in. This will definitely increase the usage but may impact on meaningful hypothesis generation at a later date. Less important details in initial stages may turn out to be of value in the future. Boffa et al. have pointed out that the National Cancer Database had not collected some important patient attributes.^2^

Observational research is not a simple alternative to controlled trials and the quality of data collected is important. The data collected should be suited to the research question, and guidelines have been published for assessing such studies.^3^ We agree with the authors that national societies should plan for the future and support the development of registries. The quantity and quality of scientific data should increase and be easily available for making informed rational decisions.
